# Supplementary figures and images for: scRNA-seq and scATAC-seq analyses highlight the role of TNF signaling pathway in chronic obstructive pulmonary disease model mice
Source: PLoS One. 2025 May 9;20(5):e0322538. doi: 10.1371/journal.pone.0322538 (PMC12063857; doi:10.1371/journal.pone.0322538)

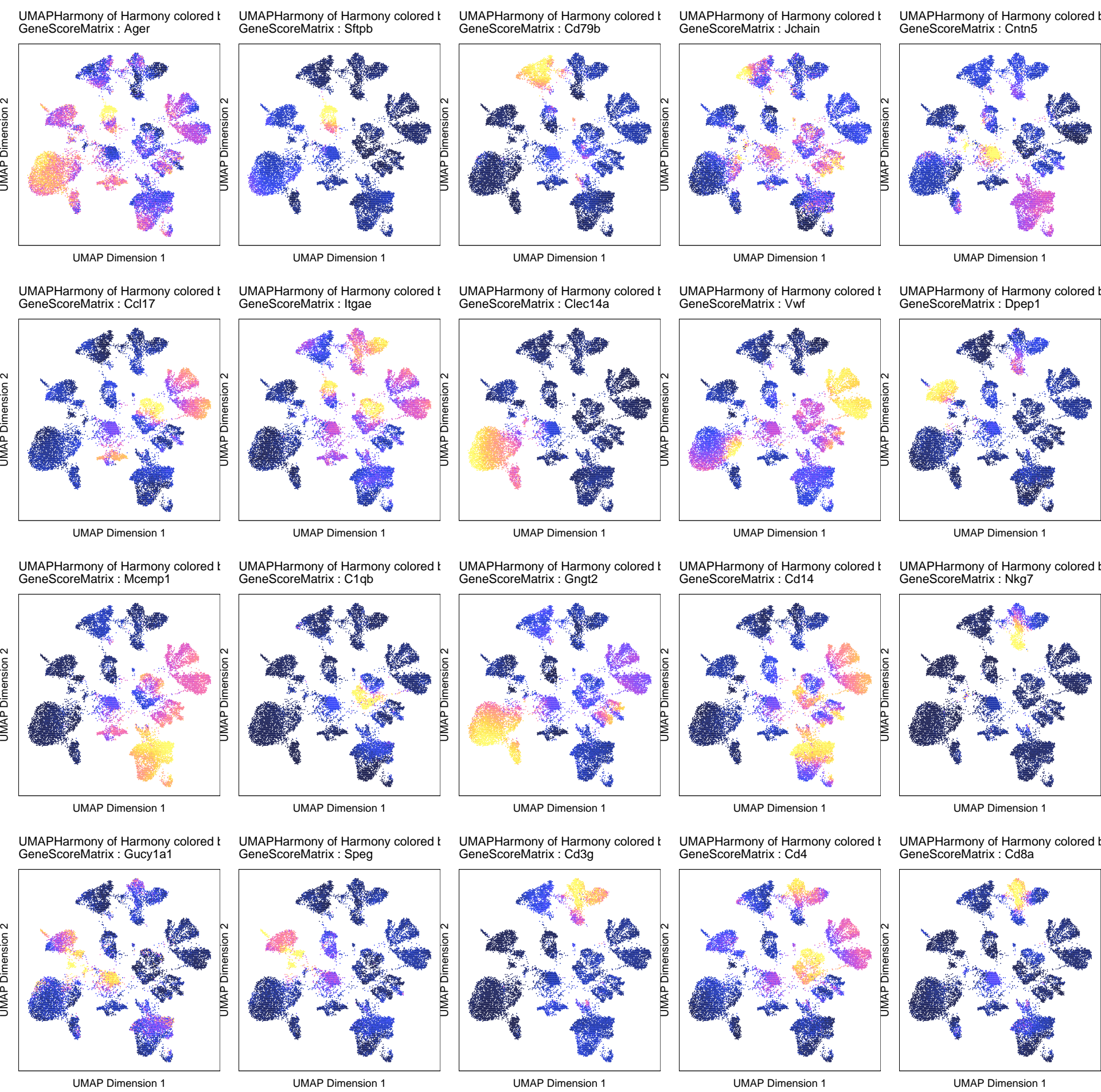

Supplement: S1 Fig — Colors from dark blue to yellow represent the gene score from low to high level. (PDF) [file pone.0322538.s001.pdf]

CellType

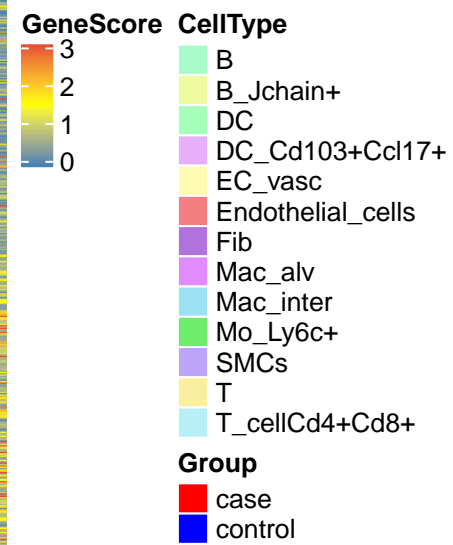

Group

Supplement: S2 Fig — Colors on the top show the different cell types while the bottom color means the case(red) and control(blue) group. (PDF) [file pone.0322538.s002.pdf]

B cell 1.05

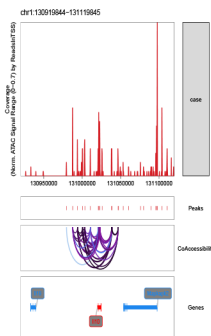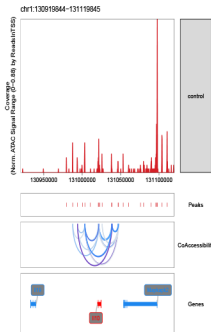

B J 2.12

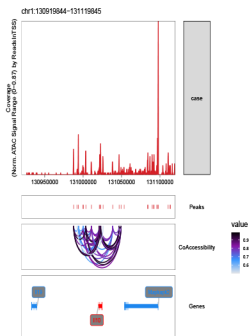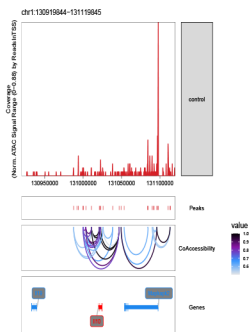

DC 2.32

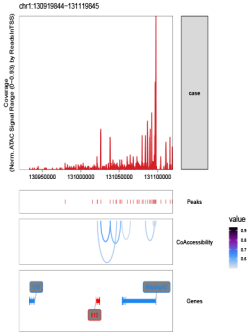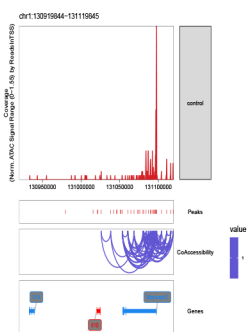

DC+

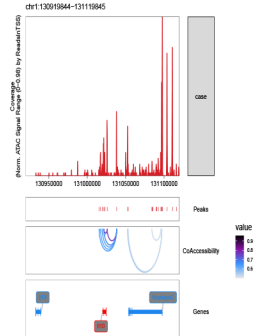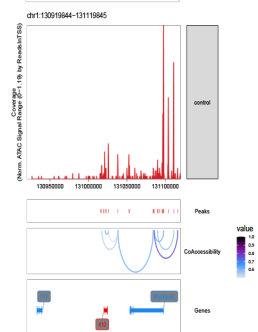

En 3

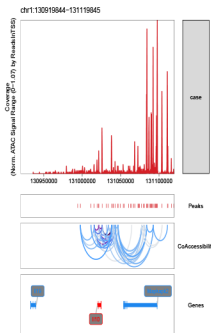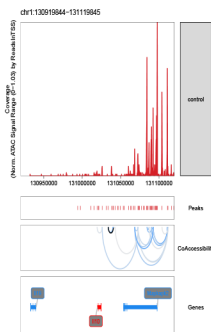

Mac-alv 1.18

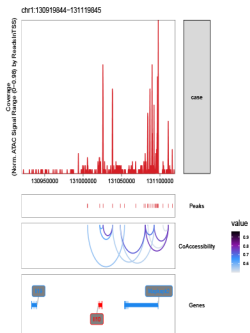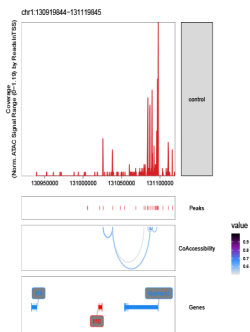

Mo 1.82

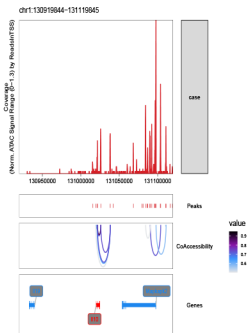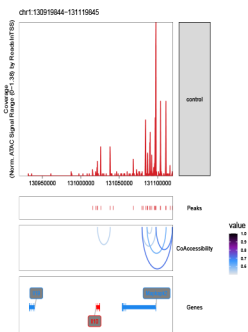

T cell

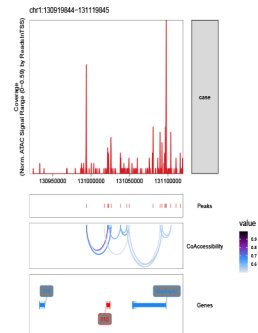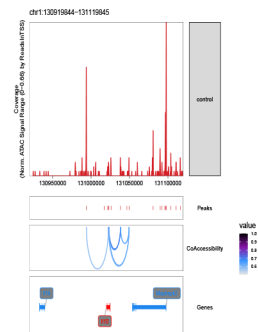

Supplement: S3 Fig — Cell types are labeled on the top of each column. Colors from grey to dark represent the accessibility levels from low to high. (PDF) [file pone.0322538.s003.pdf]

## B Jchain

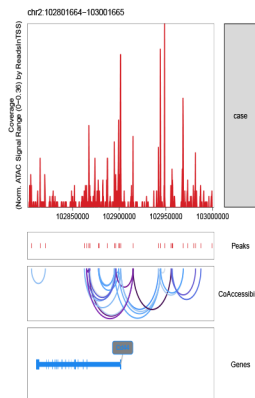

## Fib

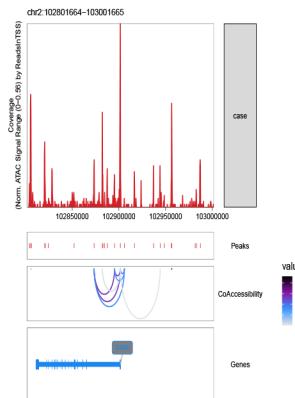

## Mac-alv

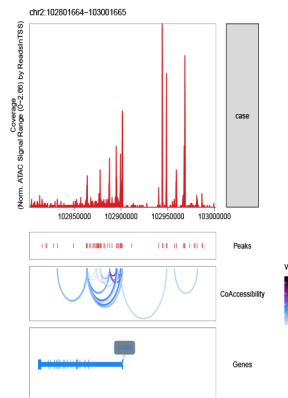

## T\_cellCd4+Cd8+ control

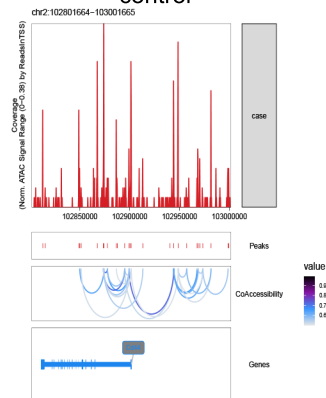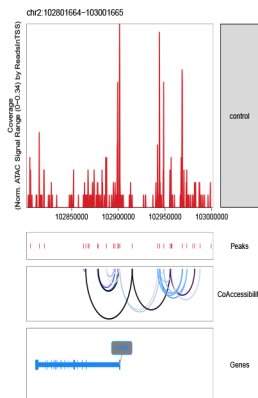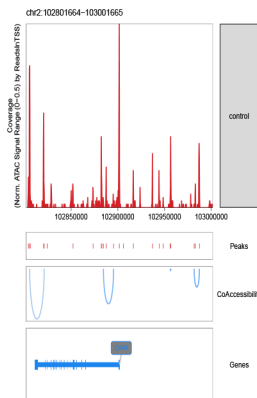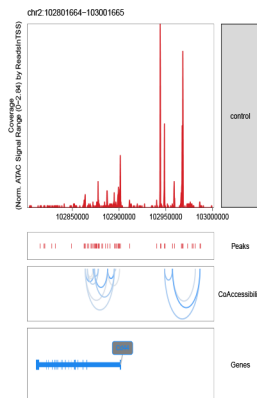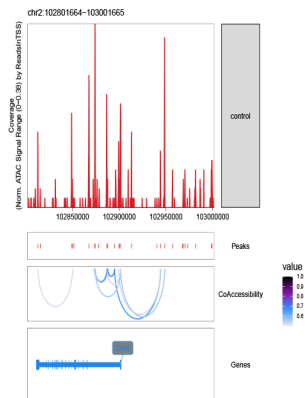

Supplement: S4 Fig — Cell types are labeled on the top of each column. Colors from grey to dark represent the accessibility levels from low to high. (PDF) [file pone.0322538.s004.pdf]

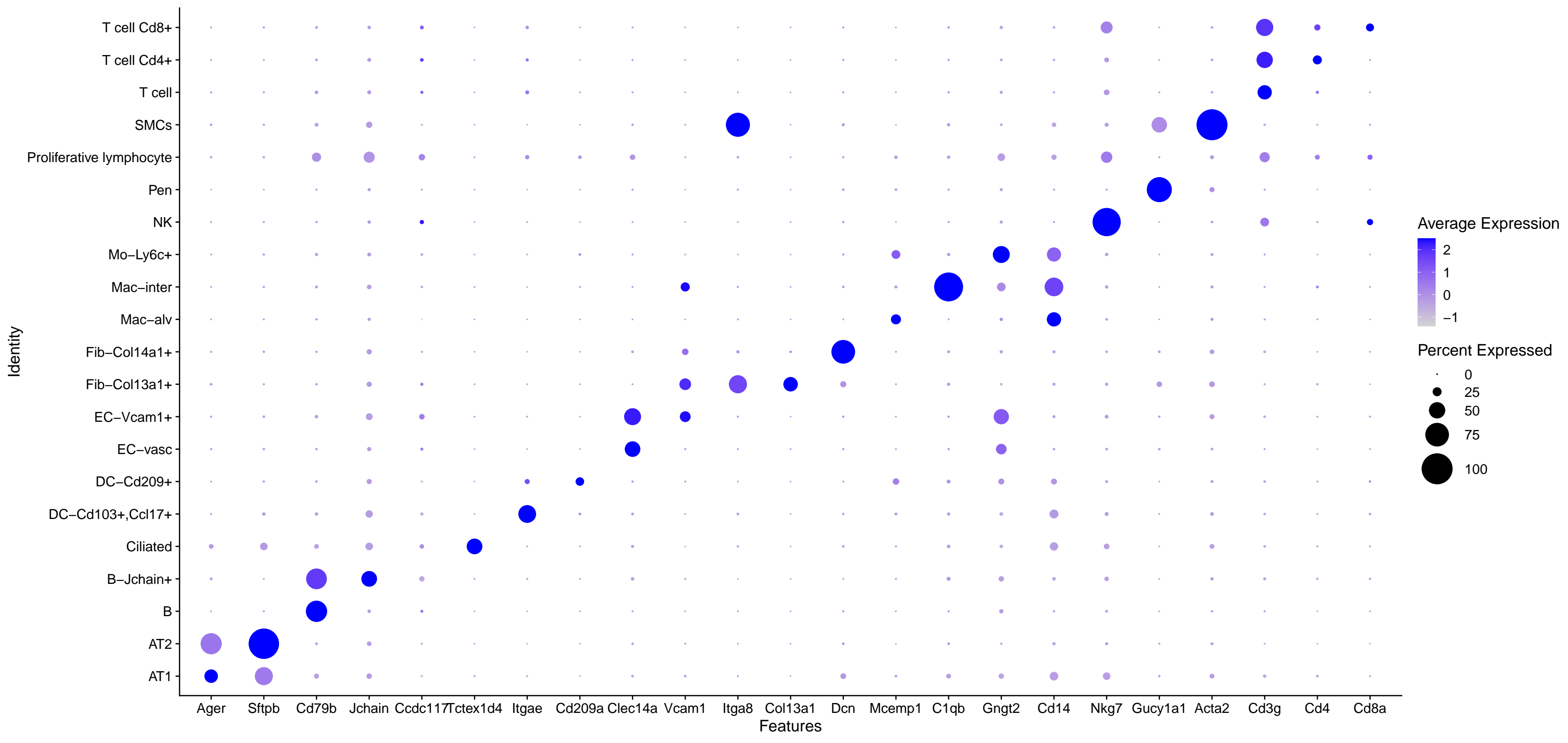

Supplement: S5 Fig — The expression of identifying markers is sometimes evident in several clusters. For each group of markers, the dot size indicates the mean fraction of cells expressing the markers. Color indicates mean expression level. (PDF) [file pone.0322538.s005.pdf]

case

control

AT1

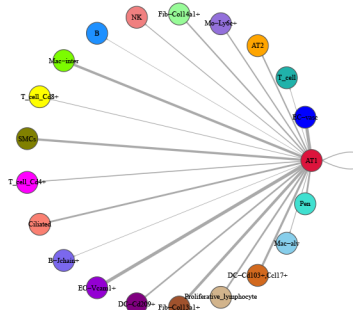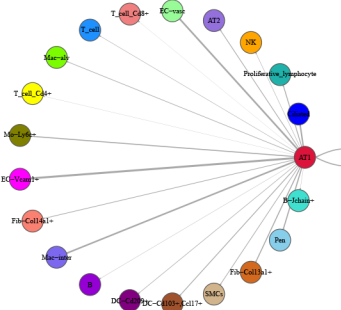

DC-Cd209

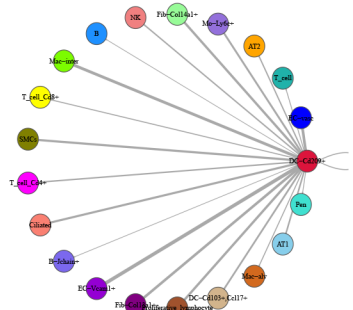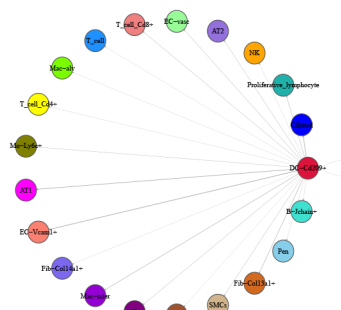

Fib-Col13a1

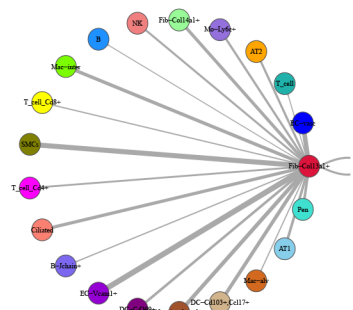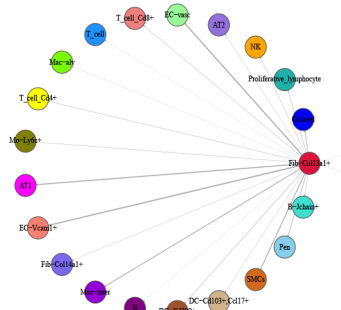

T\_cell\_Cd4

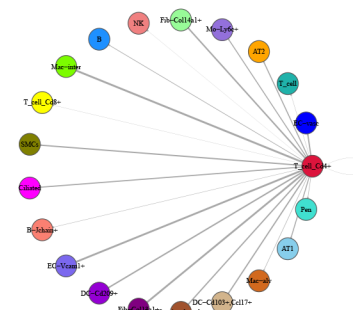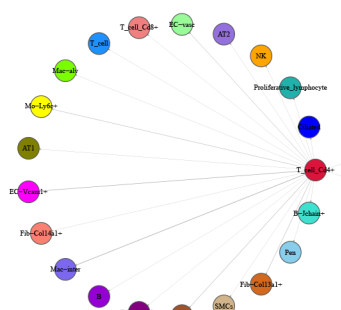

Supplement: S6 Fig — Cell-cell communication is indicated by the connected line. The thickness of the lines is positively correlated with the number of ligand-receptor interaction events. (PDF) [file pone.0322538.s006.pdf]
