# Supplementary material for: scRNA-seq and scATAC-seq analyses highlight the role of TNF signaling pathway in chronic obstructive pulmonary disease model mice
Source: PLoS One. 2025 May 9;20(5):e0322538. doi: 10.1371/journal.pone.0322538 (PMC12063857; doi:10.1371/journal.pone.0322538)
Supplement: S1 Table — (DOCX) [file pone.0322538.s007.docx]

**Supplementary Table**

**S1 Table.**  Single-cell ATAC-seq QC statistics

| **Model number** | **Estimated number of cells** | **Median fragments per cell** | **Median fraction of fragments overlapping peaks** | **Median fraction of fragments overlapping TSSs** | **Total number of reads pairs** | **Reads Pass QC** | **Called peak number** | **nCell Pass filter** | **Median fragments** |
| --- | --- | --- | --- | --- | --- | --- | --- | --- | --- |
| C57-F-1 | 1,409 | 5911 | 66.84% | 48.47% | 720,864,300 | 623,232,183 | 46,133 | 1,398 | 5,907 |
| C57-F-2 | 4897 | 4713 | 74.72% | 51.33% | 721,628,433 | 592,651,530 | 71,583 | 4,864 | 4,712 |
| C57-F-3 | 2960 | 4997 | 57.99% | 36.45% | 745,191,512 | 622,931,686 | 54,235 | 2,725 | 4,803 |
| C57-M-1 | 5664 | 3942.5 | 72.36% | 52.58% | 577,883,478 | 487,458,891 | 62,649 | 5,596 | 3,943 |
| C57-M-2 | 6448 | 4992 | 64.60% | 39.62% | 663,394,917 | 572,664,380 | 75,168 | 6,167 | 5,088 |
| C57-M-3 | 2568 | 5717 | 68.01% | 46.03% | 711,665,181 | 599,918,073 | 54,432 | 2,553 | 5,705 |

**S2 Table.**  Peak number statistics

| **Celltype** | **Peak Number** |
| --- | --- |
| AT1_control-vs-case | 30 |
| B_case-vs-control | 1333 |
| B_control-vs-case | 680 |
| B-Jchain+_case-vs-control | 46 |
| B-Jchain+_control-vs-case | 154 |
| DC_case-vs-control | 914 |
| DC-Cd103+Ccl17+_case-vs-control | 274 |
| DC-Cd103+Ccl17+_control-vs-case | 966 |
| DC_control-vs-case | 473 |
| EC-vasc_case-vs-control | 72 |
| EC-vasc_control-vs-case | 81 |
| Endothelial-cells_case-vs-control | 6563 |
| Endothelial-cells_control-vs-case | 6446 |
| Fib_case-vs-control | 2325 |
| Fib_control-vs-case | 612 |
| Mac-alv_case-vs-control | 6188 |
| Mac-alv_control-vs-case | 5247 |
| Mac-inter_case-vs-control | 1981 |
| Mac-inter_control-vs-case | 1561 |
| Mo-Ly6c+_case-vs-control | 2434 |
| Mo-Ly6c+_control-vs-case | 3497 |
| SMCs_case-vs-control | 1954 |
| SMCs_control-vs-case | 365 |
| T_case-vs-control | 59 |
| T_cellCd4+Cd8+_case-vs-control | 343 |
| T_cellCd4+Cd8+_control-vs-case | 120 |
| T_control-vs-case | 232 |
