# Supplementary material for: scRNA-seq and scATAC-seq analyses highlight the role of TNF signaling pathway in chronic obstructive pulmonary disease model mice
Source: PLoS One. 2025 May 9;20(5):e0322538. doi: 10.1371/journal.pone.0322538 (PMC12063857; doi:10.1371/journal.pone.0322538)
Supplement: Attachment — Submitted filename: Response to Reviewers.doc [file pone.0322538.s010.doc]

**Reviewer #1:**

This manuscript explores the role of the TNF signaling pathway in chronic obstructive pulmonary disease (COPD) using scRNA-seq and scATAC-seq analyses in model mice. The integration of these high-throughput techniques provides a comprehensive view of the transcriptomic and chromatin accessibility changes associated with COPD, focusing particularly on the TNF pathway's role in inflammation and immune cell function. The study is methodologically sound, and the findings are significant as they contribute to understanding the molecular mechanisms underpinning COPD, potentially guiding targeted therapies. The dual use of scRNA-seq and scATAC-seq provides a robust dataset for analyzing gene expression and regulatory elements in COPD.Detailed methodological description enhances the reproducibility of the research.Significant insights into the role of the TNF signaling pathway in COPD pathogenesis, supported by comprehensive data analysis.
**The manuscript would benefit from a more extensive review of the literature on TNF signaling in other inflammatory and pulmonary conditions to position its findings within a broader context. In the introduction, mode details should be mentioned for cancer treatment, such as referring to “Cancer treatments: Past, present, and future, 2024”.**

Response: Thank you for your comments. As you suggest, we have made revisions in our manuscript, focus on the introduction and discussion sections. We reviewed the relevant literature, and added the relevant studies on TNF signaling pathway in diseases, such as cancer (Lung cancer, Breast cancer), allergic asthmatic, and lung injury.

**Additional comparisons with other studies that have used similar methodologies in different diseases could highlight the unique contributions of this work to the field of pulmonary research. The paper should refer to other studies using scRNA seq analysis, such as “Identification of the novel exhausted T cell CD8 + markers in breast cancer, 2024” and scATAC-seq such as “Integrative Single-Cell RNA-Seq and ATAC-Seq Analysis of Human Developmental Hematopoiesis, 2021”**

Response: Thank you for your comments. We have read the reference “Integrative Single-Cell RNA-Seq and ATAC-Seq Analysis of Human Developmental Hematopoiesis, 2021”. The data analysis strategy of the reference is basically the same as our data analysis strategy, only the subheadings of each method are named differently. For example, “Upstream analysis of RNA-Seq data” mentioned in the reference corresponds to our method “RNA-Seq raw data processing”; “Upstream analysis of scATAC-Seq data” corresponding to our method “scATAC-seq data pre-processing” and “scATAC-seq quality control”; “Dimensionality reduction of scATAC-Seq data” corresponding to our method “scATAC-seq batch alignment and dimensionality reduction”; “Cell type classification” corresponding to our method “scRNA-seq cell clustering and identification”; “Differential expression analysis” corresponding to our method “DEG identification and analysis”; “Integration of scRNA-Seq and scATAC-Seq data” corresponding to our method “scATAC-seq and scRNA-seq dataset integration”.

**The manuscript presents valuable findings that add significantly to our understanding of the molecular underpinnings of COPD, particularly through the lens of the TNF signaling pathway**. **However, enhancing the literature review, providing more detailed methodological justifications, and expanding on the clinical implications and limitations of the study would improve its impact and relevance. These improvements would not only solidify the current findings but also help pave the way for future research and potential clinical applications.**

Response: Thank you for your comments. As you suggest, we reviewed the relevant literature, and supplemented the relevant research on TNF signaling pathway in lung disease. Meanwhile, in the penultimate paragraph of the discussion, we discussed the limitations of this study.

**Reviewer #2:**

1. **In the abstract section, the term scATAC should not be abbreviated at first mention.**

Response: Thank you for your comments. We have added the full name of scATAC. Please see the abstract section.

**2. In the introduction L 48, the HHIP should be given completely when first mentioned.**

Response: Thank you for your comments. We have added HHIP's full name. Please see line 49.

**3.From line 56 to L 66, the paragraph is mostly methodology and conclusion. The purpose of the study is not elaborated. The same goes to the importance of HHIP and scATAC in COPD study.**

Response: Thank you for your comments. We have added the purpose of this study. Please see line 69 to L 71.

**4. Do you think 2 mice for each group is enough for accurate experiment and data?**

Response: As you said, this study included a relatively small sample size. Indeed, We used eight mice (4 male and 4 female) to create animal models. It is a pity that relatively comprehensive experimental arrangements was not performed in the process of model building and sample collection due to feeding, transportation and other reasons.

**5. The discussion is general and mostly repeating to results. You need to signify each of the parameters studied in initiating COPD.**

Response: Thank you for your comments. As you suggest, we have made revisions in our manuscript, focus on the discussion section. Meanwhile, we have added the relevant studies on TNF signaling pathway in lung diseases.
