# Supplementary material for: scRNA-seq and scATAC-seq analyses highlight the role of TNF signaling pathway in chronic obstructive pulmonary disease model mice
Source: PLoS One. 2025 May 9;20(5):e0322538. doi: 10.1371/journal.pone.0322538 (PMC12063857; doi:10.1371/journal.pone.0322538)
Supplement: Attachment — Submitted filename: Response_to_Reviewers_auresp_2.doc [file pone.0322538.s011.doc]

**Reviewer #3:**

In this work, the authors establish and then generate from a mouse COPD model single cell ATAC-seq and RNA-seq datasets. They interrogate these data using an array of current computational biology approaches. They find that immune response cell such as B cells, mononuclear cells/ macrophages and dendritic cells are associated with COPD. They also find that TNF signaling may play a key role in the disease.
The work done, beginning with creation of the model, is thorough and the characterizations of the model are, potentially, a worthy contribution. The manuscript is generally well written. Moreover, the authors have been careful to point out the limitations of the study. **The major difficulty I have with this work is that, beyond the novel combination of approaches, the authors do not highlight the gap in knowledge that this study’s findings help fill (if any).** For example, there is previous evidence of an association of TNF signaling with COPD in the literature, so there is a need to highlight what is novel here.

Response: Thank you for your comment. As you said, we do not highlight the gap in knowledge that our study’s findings help fill. Indeed, animal models and research methods are the innovations of our study. Our study provides the data of scRNA-seq and scATAC-seq for COPD model mice. Through the joint analysis of scRNA-seq and scATAC-seq, the cell map was systematically mapped, and from the aspects of differential genes, signaling pathways and cell communication to understand the pathogenesis of COPD. TNF signaling has been reported to be associated with COPD. Our study also found that TNF signaling, which is consistent with previous studies. In the last paragraph of the introduction, the first and last paragraph of the discussion section, we describe the key points and key results of this study.

**Some suggested edits:**
**-pages need to be numbered**

Response: Thank you for your comments. We paginated our manuscript.

**-on line 198, regarding the use of the Seurat package’s FindAllMarkers function, it is not specified what test was used (for example Wilcox, t-test, DESeq2, or other)**

Response: Thank you for your comment. In our analysis using the FindAllMarkers function from the Seurat package, we used the default statistical test, which is the Wilcoxon rank-sum test (as documented in Seurat). This method identifies differentially expressed genes between clusters. We have made revisions in our manuscript.

**-on line 200, enrichKEGG from clusterProfiler could not have been used for Gene Ontology enrichment**

Response: Thank you for your comment. You are absolutely correct that the enrichKEGG function from the clusterProfiler package is specifically designed for KEGG pathway enrichment analysis and cannot be used for Gene Ontology (GO) enrichment. For our GO enrichment analysis, we actually used the enrichGO function from clusterProfiler. We acknowledge that this was not clearly described in the original manuscript, which may have caused confusion. As you suggest, we have made revisions in our manuscript.

**-on line 210, please specify the source of the addGeneIntegrationMatrix function i.e. ArchR**

Response: Thank you for your suggestion. We confirm that the addGeneIntegrationMatrix function is from the ArchR package. We agree that explicitly stating the source would enhance the clarity of the manuscript. Therefore, we have updated the text.

**-on line 265 “based” should replace “base”**

Response: Thank you for your comments. We have made revisions in our manuscript.

**-on line 286 “There are color represents” needs to be corrected**

Response: Thank you for your comments. We have made revisions in our manuscript.

**-lines 309 -312: please specify the figure numbers that are relevant here (was it Figure 3d?). Indeed in much of the Discussion section, refernces to results are made without stating what the associated Figure numbers are**

Response: Thank you for your comments. As you suggest, we have made revisions in our manuscript.

**-lines 323 et seq: Mouse gene symbols should be “Sox7, Hoxa5”, etc… rather than the human “SOX7, HOXA5”, etc.. as was stated. Moreover, gene symbols need to be italicized**

Response: Thank you for your comments. We have made revisions in our manuscript (line 323). Meanwhile, the gene symbols that appear in our manuscript are shown in italics.

**-lines 409 – 417: A number of major statements are made without the requisite literature citations**

Response: Thank you for your comments. As you suggest, We have added the relevant references.
